# 1 km land use/land cover change of China under comprehensive socioeconomic and climate scenarios for 2020–2100

**DOI:** 10.1038/s41597-022-01204-w

**Published:** 2022-03-28

**Authors:** Meng Luo, Guohua Hu, Guangzhao Chen, Xiaojuan Liu, Haiyan Hou, Xia Li

**Affiliations:** 1 Key Lab of Geographic Information Science (Ministry of Education), School of Geographic Sciences, East China Normal University, 500 Dongchuan Road, Shanghai, 200241 China; 2 Institute of Future Cities, The Chinese University of Hong Kong, Shatin, NT Hong Kong SAR

**Keywords:** Environmental impact, Projection and prediction

## Abstract

In the past decades, China has undergone dramatic land use/land cover (LULC) changes. Such changes are expected to continue and profoundly affect our environment. To navigate future uncertainties toward sustainability, increasing efforts have been invested in projecting China’s future LULC following the Shared Socioeconomic Pathways (SSPs) and/or Representative Concentration Pathways (RCPs). To supplements existing datasets with a high spatial resolution, comprehensive pathway coverage, and delicate account for urban land change, here we present a 1-km gridded LULC dataset for China under 24 comprehensive SSP-RCP scenarios covering 2020–2100 at 10-year intervals. Our approach is to integrate the Global Change Analysis Model (GCAM) and Future Land Use Simulation (FLUS) model. This dataset shows good performance compared to remotely sensed CCI-LC data and is generally spatio-temporally consistent with the Land Use Harmonization version-2 dataset. This new dataset (available at 10.6084/m9.figshare.14776128.v1) provides a valuable alternative for multi-scenario-based research with high spatial resolution, such as earth system modeling, ecosystem services, and carbon neutrality.

## Abstract (summary)

**Table:** 

Measurement(s)	Land Use and Land Cover Change
Technology Type(s)	computational modeling technique
Factor Type(s)	Shared Socioeconomic Pathways scenarios • Representative Concentration Pathways scenarios • land use and land cover change
Sample Characteristic - Environment	Land
Sample Characteristic - Location	China

## Background & Summary

Land use/land cover (LULC) plays a crucial role in the interactions between the human system and the Earth system^1^, which relates directly to a wide range of issues that involve big stakes, e.g., biodiversity^2^, energy balance^3^, carbon cycle^2^, hydrologic cycle^4^, and climate extremes^5^. In this regard, LULC in China has been undergoing dramatic changes in the past few decades, with nationwide and worldwide social-environmental consequences^1,6,7^. For instance, since the “Reform and Opening-up” in 1978, China’s rapid urban growth has prevailed by invading a large proportion of croplands^8–10^. However, after implementing afforestation and reforestation programs, China has shown a significant vegetation greening trend, contributed mainly by forests (42%) and croplands (32%)^1,11^. These complicated changes are subject to the influences of a variety of social and economic factors. Further investigating and predicting future LULC in China is of vital importance for future land use policy decisions and sustainable management of ecosystems. This could provide the crucial information to balance the anthropogenic climate change and social-economic development.

Combining socio-economic scenarios, emission pathways, and diverse sectoral information in a unified framework can be used to assess future LULC under different policies and global mitigation targets. Integrated Assessment Models (IAMs) are commonly used to quantify outcomes of LULC under different Shared Socioeconomic Pathways (SSPs, representing alternative socio-economic developments)^12^ and Representative Concentration Pathways (RCPs, representing greenhouse gas concentration trajectories)^13^, such as the Asian-Pacific Integrated Model/computable general equilibrium (AIM/CGE)^14^, Integrated Model to Assess the Global Environment (IMAGE)^15^, and Global Change Analysis Model (GCAM)^16^. Among them, GCAM, an open-source global integrated and multi-sector model adopted by the Intergovernmental Panel on Climate Change (IPCC)^17^, has been widely used to project future LULC under diverse socioeconomic and emission scenarios at both regional and global scales^18–20^. GCAM V5.2 explicitly incorporates modules of water supply and demands which is vital in the agriculture and land use sectors^21^. Besides, GCAM V5.2 also considers the assumptions of water technological advancements under different SSPs, which has significant impacts on water demands in a water-constrained world^22^.

Mapping spatially explicit LULC patterns at a high spatial resolution is important for analyzing the local spatial details of LULC and understanding the local interactions among human activities and ecological processes in the alternative future^23^. Some research pointed out that LULC data with coarse spatial resolution could ignore a large proportion of small urban patches, with a severe underestimation of urban land area and urban growth^24^. By contrast, LULC data with a fine spatial resolution (like 1-km) could provide more necessary spatial details and accurately reflect the heterogeneous spatial characteristics of LULC^24^. However, the LULC data produced by IAMs are typically in the subregion levels (e.g., the regional, agroecological or water-basin levels)^16,25,26^ or with coarse spatial resolution^14,27,28^. Combining IAMs under global scenarios with spatially-explicit LULC models at a coherent framework provides a feasible scheme to project future LULC with a finer spatial resolution^18–20^. For example, the Future Land Use Simulation (FLUS) model has been used to generate spatially-explicit LULC data by combining IAMs^19,24^, which can simulate high-spatial-resolution LULC change with generally higher accuracy than the single neural network-based cellular automata (CA) model^29,30^, the Conversion of Land Use and its Effects at Small regional extent (CLUE-S)^31^, and other models^24,32,33^.

Recently, a set of SSP-RCP frameworks have been proposed^34,35^ to describe potential pathways under diverse socio-economic and emission conditions. However, comprehensive assessments of China’s LULCs under full combinations of SSP and RCP scenarios with high resolutions remain to be conducted. It can facilitate the thorough analysis of our uncertain future under challenges of mitigation and adaptation^35^ and is also crucial for the net-zero emission research^36,37^. Some studies produced the LULC projections under the combinations of SSP and RCP scenarios at a coarse spatial resolution, such as the 0.5-degree LULC data projected by AIM/GEC^14^ and five arc-minute gridded LULC data produced by IMAGE^27^. In contrast, some other studies generated the future LULC data with a high spatial resolution but only covered very limited scenarios. For example, Dong *et al*.^19^ developed 1-km resolution LULC data in China using the integrated GCAM and FLUS model. Cao *et al*.^20^ spatialized global LULC data at a 1-km resolution based on the integrated GCAM and CA model. However, missing some important scenarios could hinder the applications in ecological and hydrological modelling^38^,^39^. For example, SSP5-RCP1.9, which is a combination of a strict climate target and a fossil-fueled development scenario, may be required to represent extreme conditions of human activities in the modelling. Projecting high-spatial-resolution LULC with all possible combinations of SSP and RCP, enables a comprehensive analysis of LULC under different socio-economic assumptions and mitigation policies and can support a deep understanding of local LULC dynamics with more spatial details.

In addition, the future urban land change has not been well considered in the existing future LULC data. Urban land is a key driver for many environmental and societal changes across scales^40^ and is also crucial for studying LULC projections^32,41^. Some models currently assume that there is no urban land change in the future, such as GCAM^42^. However, there has been a dramatic change of global urban land in the past decades^43^, implying that assuming a constant coverage of the urban land in the future is unrealistic^24,40^. Some other studies projected urban shrinkage simply based on the empirical relationship between urban land, Gross Domestic Product (GDP), population, and other factors^19,27^. Nevertheless, the decrease of the urban population does not necessarily lead to numerous land conversions from urban to non-urban areas^44,45^, especially in China^46^. Recently, Chen *et al*. (2020a) developed a 1-km gridded dataset of globally future urban land expansion under SSPs, which provides a more reasonable projection of future urban land change. This dataset offers us a great opportunity to calibrate urban land change in LULC predictions.

The overarching goal of this study is to develop a high-resolution gridded LULC dataset in China under a full SSP/RCP matrix from 2020 to 2100, where the urban land change is well incorporated. First, we generated the LULC projections for China under all possible combinations of SSP and RCP using GCAM at the water-basin level. Then, we used the urban land dataset developed by Chen *et al*. (2020a) to calibrate the urban land demand (the projected total area of urban) projected by GCAM. Finally, we downscaled the water-basin-level LULC projections to 1-km grids using the FLUS model. This newly gridded dataset fills the gap between high spatial resolution and limited scenarios in the current LULC predictions, which can enhance climate change research under diverse socioeconomic and emission assumptions, provide support for making policies to limit global warming to below 2 °C or 1.5 °C by 2100, the target of the Paris agreement^36^ and help reduce the uncertainties of the Earth system modelling. It will be helpful to those researches focusing on individual socio-economic or emission conditions. Besides, this dataset will be valuable for wide unified and comparable multi-scenario-based research, such as ecosystem services^47^, biodiversity^48^,soil erosion by water^49^, and carbon neutrality in China.

## Methods

### Overall framework

**Fig. 1:**
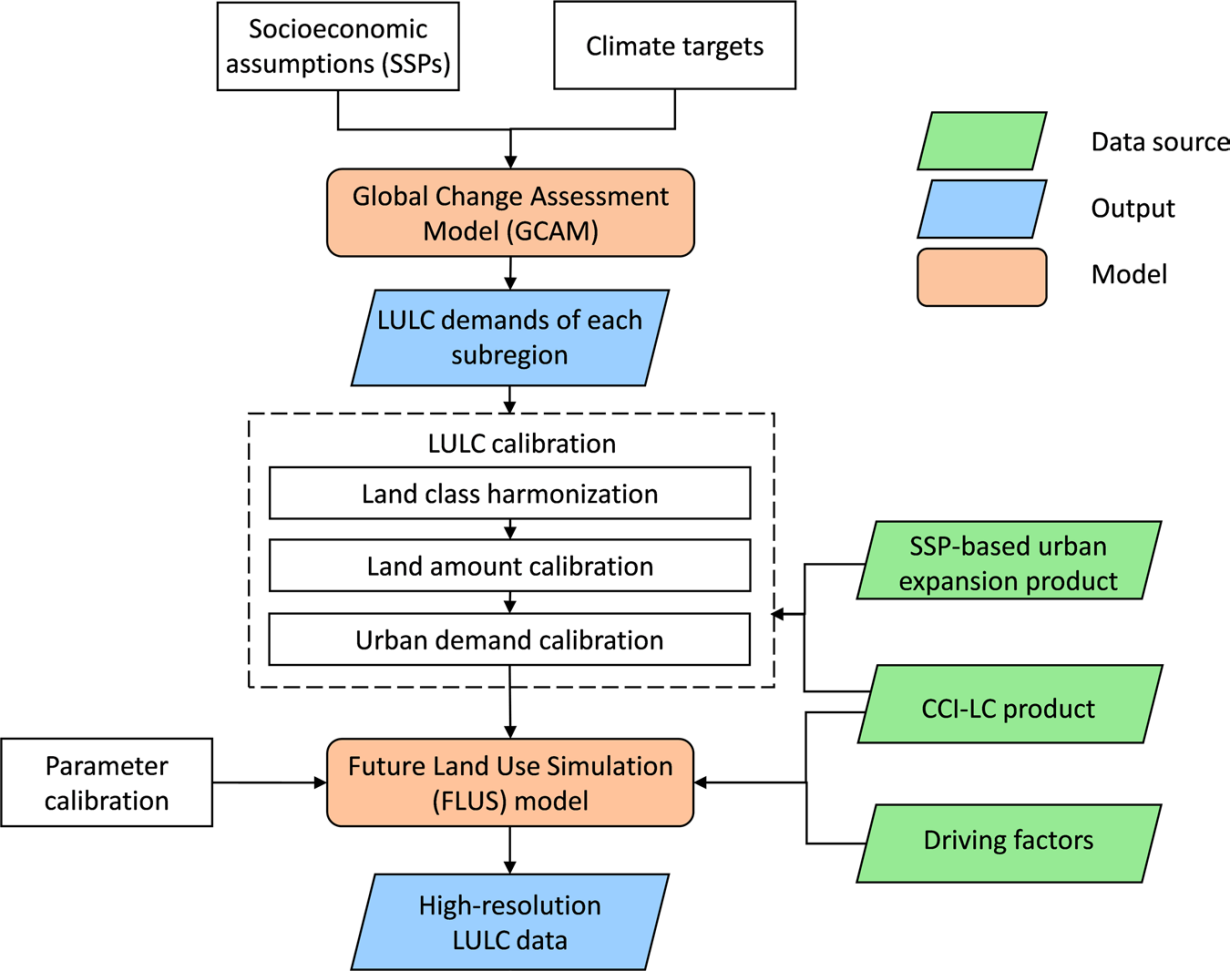
The methodological framework of the integrated GCAM-FLUS model for producing high-resolution future LULC dataset in China. The green parallelograms, blue parallelograms and orange rounded rectangle represent the data source, output data and model used to generate the LULC predictions, respectively.

Figure 1 shows the methodological framework of the integrated GCAM-FLUS model for producing high-resolution LULC dataset in China. First, we used the GCAM model to project the LULC demands of China from 2020 to 2100 under all possible SSP-RCP scenarios (24 scenarios in total, see Table 1) with a 10-year interval at a regional scale. Second, we harmonized the LULC types with a reclassification scheme (see Table 2) and calibrated the land demands of GCAM-generated LULC data based on the historical LULC data collected from the European Space Agency Climate Change Initiative (CCI-LC)^50^. Further, we calibrated the urban land demands using a well-validated future urban expansion dataset under SSPs produced by Chen *et al*. (2020a), freely downloaded from http://www.geosimulation.cn/GlobalSSPsUrbanProduct.html. Finally, we integrate comprehensive SSP-RCP with a land use model (FLUS) to downscale the GCAM-based LULC data into 1-km spatial resolution.

**Table 1:** SSP by RCP scenarios matrix and the coupled scenarios covered in this study.

		Socioeconomic assumptions				
		SSP1	SSP2	SSP3	SSP4	SSP5
Radiative forcing (Wm^−2^)	1.9	×	×			×
	2.6	×	×		×	×
	3.4	×	×	×	×	×
	4.5	×	×	×	×	×
	6.0			×		×
	Baseline	×	×	×	×	×

**Table 2:** The LULC reclassification scheme in this study.

LULC types in this study	GCAM LULC types	CCI-LC LULC types	LUH2 LULC types
Urban	Urban land	Urban	Urban
Cropland	Corn		
	Fiber crop		
	Fodder grass		
	Misc crop		
	Oil crop		
	Other grain	Cropland_rainfed	C3ann
	Palm fruit	Cropland_rainfed_herbaceous_cover	C3per
	Rice	Cropland_rainfed_tree_or_shrub_cover	C4ann
	Root tube	Cropland_irrigated	C4per
	Sugar crop	Mosaic_cropland	C3nfx
	Wheat	Mosaic_natural_vegetation	
	Other arable land		
	Biomass grass		
	Biomass forest		
	RootTuber		
Grassland	Managed pasture	Cosaic_herbaceous	
	unmanaged pasture	Grassland	
	GrasslandGrassland	Lichens_and_mosses	Pastr
Shrub	Shrub	Shrubland	Gange
		Shrubland_evergreen	
		Shrubland_deciduous	
		Shrub_or_herbaceous_cover_flooded	
Forest		Tree_broadleaved_evergreen_closed_to_open	
		Tree_broadleaved_deciduous_closed_to_open	
		Tree_broadleaved_deciduous_closed	
		Tree_broadleaved_deciduous_open	
	Managed forest	Tree_needleleaved_evergreen_closed_to_open	Primf
	Unmanaged forest	Tree_needleleaved_evergreen_closed	Secdf
		Tree_needleleaved_evergreen_open	
		Tree_needleleaved_deciduous_closed_to_open	
		Tree_needleleaved_deciduous_closed	
		Tree_needleleaved_deciduous_open	
		Tree_mixed	
		Mosaic_tree_and_shrub	
		Tree_cover_flooded_saline_water	
Water	None	Water	None
Bareland		Sparse_vegetation	
		Sparse_shrub	
	Rock, Desert	Sparse_herbaceous	Primn
	Tundra	Tree_cover_flooded_fresh_or_brakish_water	Secdn
		Bare_areas	
		Bare_areas_consolidated	
		Bare_areas_unconsolidated	
Ice	Ice	Snow_and_ice	None

The 24 SSP-RCP scenarios (listed in Table 1) are composed of five baseline scenarios that do not include any climate mitigation policy and 19 combined scenarios. In this study, the climate conditions are represented by three RCP levels (2.6, 4.5, 6.0 Wm^−2^) and two additional forcing levels (1.9 and 3.4 Wm^−2^). The level of 8.5 Wm^−2^ is not included because the forcing levels of all five baseline scenarios in our simulations are lower than 8.5 Wm^−2^. The two additional forcing levels are related to current policy targets^36^ and belong to 1.5 °C and 2 °C scenarios^14,38^, which play a key role in reducing the impact and risks of climate change^51,52^.

### Projection of future regional LULC demands

GCAM, a market-equilibrium dynamic-recursive model, well represents the interactions of five sub-systems: energy, water, agriculture and land use, economy, and climate^25^. GCAM is one of the marker models used to quantify SSP and RCP scenarios and has been widely used to analyze future LULC changes under different scenarios. In the newly released GCAM v5.2, the land-use module subdivides 32 global geo-political regions into the water-basin levels, and there are totally 24 subregions in China (Figure S1). GCAM uses a logit model to represent the sharing of the economic decisions for land use in one region. Thus, there is a distribution of profit behind each competing land use. GCAM also uses a nesting strategy to reflect the differences in alternatives through different LULC types with logit exponents. Besides, GCAM implements both SSP and RCP scenarios. This study used GCAM v5.2 to project the water-basin level demands of different LULC types from 2020 to 2100 with a 10-year interval under the 24 scenarios.

### Calibration of LULC types and land demands

There are some inconsistencies of LULC types between the output of GCAM and the initial LULC map (i.e., CCI-LC) used for spatial downscaling. GCAM mainly includes nine LULC types composed of Cropland, Forest, Pasture, Grassland, Shrub, Urban land, Tundra, Rock, Ice and Desert, and Biomass. By contrast, CCI-LC mainly includes Cropland, Grassland, Shrub, Forest, Bareland, Ice, Water and Urban areas. Besides, there are considerable differences in land areas between the GCAM-derived future land demands and CCI-LC dataset. These inconsistencies may cause significant errors and uncertainties in the future LULC simulations. Therefore, we first built a reclassification scheme composed of 8 LULC types (Table 2) and used it to harmonize the LULC types of these two datasets. Then, we further adjusted the GCAM-derived future land demands by:

1 $$A{(t+1)}^{k}=\left\{\begin{array}{c}\left(1+\frac{A{\left(t+1\right)}_{GCAM}^{k}-A{\left(t\right)}_{GCAM}^{k}}{A{\left(t\right)}_{GCAM}^{k}}\right)\times A{(t)}_{CCI-LC}^{k}\quad t=2010\\ \left(1+\frac{A{\left(t+1\right)}_{GCAM}^{k}-A{\left(t\right)}_{GCAM}^{k}}{A{\left(t\right)}_{GCAM}^{k}}\right)\times A{(t)}^{k}\quad 2020\le t\le 2100\end{array}\right.$$

where $A{(t+1)}^{k}$ is the calibrated area of type *k* at year *t* + 1, $A{\left(t+1\right)}_{GCAM}^{k}$ is the area of type *k* at year *t-1* calculated by GCAM, and $A{(t)}_{CCI-LC}^{k}$ is the area of type *k* at year *t* from the CCI-LC data.

### Calibration of urban demand

GCAM assumes that the future urban demands remain constant, which is unreasonable^40^ and needs to be re-adjusted. Chen *et al*. (2020a) generated a 1-km future urban land expansion dataset based on the established relationships between urban land demand, GDP and urbanization rate. This dataset has a much higher spatial resolution which could capture more spatial details of urban land patterns than other urban expansion data and shows excellent performance in terms of the Figure of Merit (FoM)^32^. In addition, this urban land dataset considers more reasonable situations in the urban shrinkage stage^45,46^. In this study, we used this urban land dataset to calibrate future GCAM-derived urban demands. We first calculated the urban areas of 24 subregions under different SSP scenarios based on this dataset, and used it to update the future urban demands of GCAM-derived LULC data. Then, we used the methods developed by Li *et al*. ^24^ to adjust the areas of the remaining LUCCs proportionally. Equation 2 describes how we deduct the demand of urban encroachment in other LULC types.

2 $${A}_{adjust}{(t)}^{k}=\left(A{(t)}^{U}-A{(0)}^{U}\right)\times {H}_{n}^{k\to U}$$

where ${A}_{adjust}{(t)}^{k}$ is the area of type *k* encroached by urban at year *t*, $A{(t)}^{U}A$ is the projected area of urban land (U) for year *t*, $A{(0)}^{U}$ is the original area of urban in 2010, n is the number of LULC types (except urban, water and ice), and ${H}_{n}^{k\to U}$ is the empirical proportion of land loss encroached by urban of type k and the sum of ${H}_{n}^{k\to U}$ of all n types is 1.

In this study, we assumed that urban could encroach other LULC types except for water and ice, and the urban demand under the same SSP scenario is identical. We used the CCI-LC data in 2000 and 2010 to calculate the proportion of land loss due to the urban encroachment on each LULC type and derive ${{\rm{H}}}_{{\rm{n}}}^{{\rm{k}}\to {\rm{U}}}$ for each subregion (Table 3). Then we combined ${{\rm{H}}}_{{\rm{n}}}^{{\rm{k}}\to {\rm{U}}}$ and aforementioned urban land dataset to adjust the demands of other LULC types in the GCAM-derived LULC data.

**Table 3:** The calculated ${{\rm{H}}}_{{\rm{n}}}^{{\rm{k}}\to {\rm{U}}}$ for different LULC types in 24 subregions of China.

${{\bf{H}}}_{{\bf{n}}}^{{\bf{k}}\to {\bf{U}}}$	LULC type				
Region code	Forest	Shrub	Grassland	Cropland	Bareland
1	0.55	0.10	0.04	0.06	0.26
2	0.36	0.40	0.01	0.00	0.24
3	0.80	0.09	0.02	0.05	0.04
4	0.56	0.22	0.02	0.00	0.20
5	0.68	0.18	0.01	0.06	0.07
6	0.76	0.21	0.00	0.03	0.00
7	0.82	0.17	0.00	0.00	0.00
8	0.88	0.10	0.01	0.02	0.00
9	0.75	0.23	0.00	0.00	0.01
10	0.47	0.34	0.01	0.00	0.18
11	0.00	0.00	0.00	0.00	1.00
12	0.25	0.25	0.00	0.00	0.50
13	0.92	0.00	0.05	0.03	0.00
14	0.93	0.05	0.01	0.01	0.00
15	0.85	0.10	0.01	0.04	0.00
16	0.88	0.06	0.00	0.06	0.00
17	0.29	0.67	0.00	0.00	0.04
18	0.95	0.04	0.00	0.01	0.00
19	0.92	0.01	0.03	0.05	0.00
20	0.88	0.04	0.02	0.07	0.00
21	0.90	0.06	0.01	0.03	0.00
22	0.78	0.06	0.00	0.17	0.00
23	0.02	0.01	0.12	0.83	0.03
24	0.02	0.01	0.12	0.83	0.03

### Downscaling of regional LULC projections

Based on the GCAM-derived LULC demands calibrated through the steps abovementioned, we used the FLUS model to simulate the future LULC of China at 1-km resolution. FLUS integrates the top-down system dynamics and bottom-up CA and can explicitly simulate the spatial trajectories of multiple LULC types^33^. The first part of FLUS aims to train and estimate the occurrence probabilities of LULC on a specific grid cell based on artificial neural networks (ANN). Specifically, we first collected the CCI-LC data in 2010 and 15 driving factors (shown in Table 4) as the training data. The driving factors were mainly selected based on relevant studies and can reflect different heterogeneous characteristics (i.e., climate, soil, topography, population, economics, transportation, etc.) related to LULC^24,32,33^. All these driving factors are reprojected into 1-km × 1-km grids with a spatial reference of the Albers equal-area conic projection. Then we trained the ANN model based on a 1% uniform sample rate for each subregion and used the trained model to estimate the occurrence probabilities (OP, determined by the characteristics of each pixcel) of each pixel. The adopted ANN model has one input layer, one hidden layer with 10 neurons and one output layer in this study. Each neuron of the input layer is associated with a driving factor, and that of the output layer corresponds to OP for a specific LULC type. The sigmoid activation function was used for the hidden layer. The second part of the model, CA, considers OP, conversion cost, neighborhood condition and competition among the different LULC types to estimate the combined probability for LULC conversion. In this step, the LULC type with a higher OP estimated by the previous step is more likely to be predicted as the target LULC type. In contrast, those with a relatively lower OP can still be converted based on the roulette selection mechanism. During the allocation stage, we adopted several assumptions: first, urban expansion is irreversible; second, water and ice are not involved in LULC conversion; and third, bareland can only be infringed by urban or stay unchanged in the future, considering that GCAM cannot project the bareland change in the future and the Bareland can only change due to the urban expansion (see the previous section). Under these assumptions, combined with CCI-LC data in 2010 as an initial LULC map, we used the FLUS model to produce 1-km LULC dataset in China from 2020 to 2100.

**Table 4:** Specifications of the 15 driving factors used during the downscaling process.

Spatial Variable	Year	Resolution	Data Source
Annual mean temperature	Climatological (1970–2000)	0.5′	WorldClim v2.0(http://www.worldclim.org/)
Annual precipitation			
Soil quality (oxygen availability to roots)	2008	5′	Fischer *et al*.^59^
Soil quality (excess salts)			
Soil quality (workability)			
DEM	1996	1 km	GTOPO30 (https://www.usgs.gov/centers/eros/science/usgs-eros-archive-digital-elevation-global-30-arc-second-elevation-gtopo30)
Slope			
POP	2010	100 m	Andrea E. Gaughan *et al*.^60^
GDP	2010	1 km	Matti Kummu *et al*.^61^
Distance to rivers and lakes	2015	1 km	Resource and Environment Data Cloud Platform (http://www.resdc.cn/)
Distance to main roads			
Distance to highway			
Distance to railway			
Distance to airports	2010	1 km	Huang *et al*.^62^
Distance to urban centers	2014	1 km	United Nations, Department of Economic Social Affairs, Population Division (2014)

## Data Records

The generated LULC dataset with 1-km spatial resolution and 10-year time step from 2020–2100 covers 24 SSP-RCP scenarios in total (Table 2). The dataset is publicly available in 10.6084/m9.figshare.14776128.v1^53^ and http://www.geosimulation.cn/. All the data is stored in a commonly-used geotiff format with a spatial reference of the Albers equal-area conic projection, which can be easily accessed by ARCGIS, ENVI, MATLAB, etc. For the file naming and structure, all the files with the same SSP-RCP scenarios were grouped into the same folders with the name of “SSP-RCP” and each geotiff file is named as “SSP_RCP_Year.tif”, where “SSP” and “RCP” denote the SSP and RCP scenarios, and “Year” denotes the year of the LULC data. For example, the file storing the LULC data under SSP1-RCP1.9 in 2100 is named as “SSP1-RCP19_2100.tif”. Taking “SSP1-RCP19_2100.tif” as an example, Figure S2 shows the 2100 LULC spatial distributions under SSP1-RCP1.9. In each geotiff file, different integer values represent different LULC types: 1 Urban; 2 Cropland; 3 Grassland; 4 Shrub; 5 Forest; 6 Water; 7 Bareland; and 8 Ice.

## Technical Validation

We used the CCI-LC data in 2000 to train the FLUS model and then used the CCI-LC data in 2010 to evaluate the reliability of our downscaled dataset. We also compared our gridded dataset with the widely-used Land-use Harmonisation (LUH2) data at a 0.25 degree resolution^54^, used in the Coupled Model Inter-comparison Project Phase 6 (CMIP6)^55^. Specifically, we chose all the overlapping scenarios between our dataset and LUH2, including SSP1-RCP1.9, SSP1-RCP1.6, SSP2-RCP4.5, SSP4-RCP3.4, and SSP5-RCP3.4 for comparison. In addition, considering the inconsistency in LULC types between our dataset and LUH2, we re-grouped the LUH2 data into five broad LULC types: Cropland, Forest, Bareland, Grassland, and Urban.

To quantitatively assess the simulated LULC during the downscaling process, we calculated the overall accuracy and the Cohen’s Kappa coefficient of each subregion by validating our dataset against the CCI-LC data. Compared with the Kappa coefficient, FoM can avoid overestimating the accuracy and has been demonstrated to effectively evaluate the accuracy and has been demonstrated to be effective to evaluate the accuracy of simulating LULC changes^56,57^. Therefore, we further used the FoM metrics to assess the consistencies between the simulated LULC and remote sensing data (CCI-LC). Specifically, FoM represents the ratio of the correct predicted change to the sum of the observed and predicted change:

3 $${\rm{FoM}}=\frac{{\rm{B}}}{{\rm{A}}+{\rm{B}}+{\rm{C}}+{\rm{D}}}$$

where A represents the false area where the observed change is predicted as persistence, B represents the correct area where the observed change is predicted as change correctly, C represents the false area where the observed change is predicted as a change in the wrong LULC type, and D represents the false area where the observed persistence is predicted as change. Its value ranges from 0 to 1, and larger FoM represents a better performance on LULC simulations.

In addition, we used the Pearson correlation coefficient and root mean square difference (RMSD) to assess the spatio-temporal consistencies between our gridded LULC dataset and LUH2 data during 2020–2100.

### Validation of the downscaling process

The statistical accuracy metrics of the LULC simulations of 2010 in each subregion and the whole of China are shown in Table 5. The Kappa coefficient ranges from 0.43 to 0.75 and the overall accuracy ranges from 0.66 to 0.88 across different subregions. These two metrics have values of 0.64 and 0.79 for China. FoM varies from 0.10 to 0.17 in different subregions, and has a value of 0.13 for China. This result is identical to existing studies which showed that the FoM values were usually in the range of 0.1 to 0.3, due to the path-dependent effects^32,33,58^. The confusion matrix of the simulated LULC compared to CCI-LC data in 2010 shows that the number of mis-classified pixels is small (Table 6). These results demonstrate that the simulated LULC have a good agreement with CCI-LC in 2010.

**Table 5:** The statistical accuracy metrics of LULC simulations of 2010 in each subregion as well as the whole of China.

Region code	FoM	Kappa	Overall Accuracy	Area proportion (%)
1	0.13	0.61	0.87	0.34
2	0.11	0.71	0.85	11.66
3	0.12	0.73	0.82	9.38
4	0.10	0.65	0.75	0.96
5	0.13	0.61	0.74	3.45
6	0.10	0.52	0.68	0.60
7	0.12	0.57	0.74	3.61
8	0.10	0.67	0.80	5.17
9	0.14	0.66	0.79	8.81
10	0.15	0.60	0.80	16.43
11	0.08	0.52	0.78	1.02
12	0.11	0.62	0.86	4.27
13	0.14	0.73	0.85	0.39
14	0.14	0.73	0.83	20.93
15	0.14	0.49	0.71	4.24
16	0.10	0.47	0.72	0.77
17	0.11	0.62	0.83	3.41
18	0.09	0.63	0.80	0.74
19	0.12	0.69	0.79	0.44
20	0.15	0.61	0.74	1.74
21	0.12	0.63	0.80	1.41
22	0.14	0.54	0.76	0.23
all	0.13	0.66	0.82	100.00

**Table 6:** Confusion matrix of the simulated LULC compared to CCI-LC in 2010.

	Actual land use pixels in 2010										
Simulated land use pixels in 2010	LULC type	Cropland	Forest	Bareland	Grassland	Water	Urban	Shrub	Ice	Total	User’s Accuracy
	Cropland	22090	2436	284	2036	189	474	109	2	27620	0.8
	Forest	2373	14393	3	613	38	8	88	2	17518	0.82
	Bareland	319	6	16454	2350	36	4	57	55	19281	0.85
	Grassland	2119	607	2236	21018	75	43	68	68	26234	0.8
	Water	180	44	39	59	1476	19	35	0	1852	0.8
	Urban	302	36	8	27	9	406	6	0	794	0.51
	Shrub	129	146	64	100	27	4	312	0	782	0.4
	Ice	1	1	68	84	0	0	0	475	629	0.76
	Total	27543	17688	19280	26098	1875	958	670	628	94710	
	Producer’s Accuracy	0.8	0.81	0.85	0.8	0.79	0.42	0.46	0.79		

Overall accuracy = 0.81; Kappa coefficient = 0.75.

**Fig. 2:**
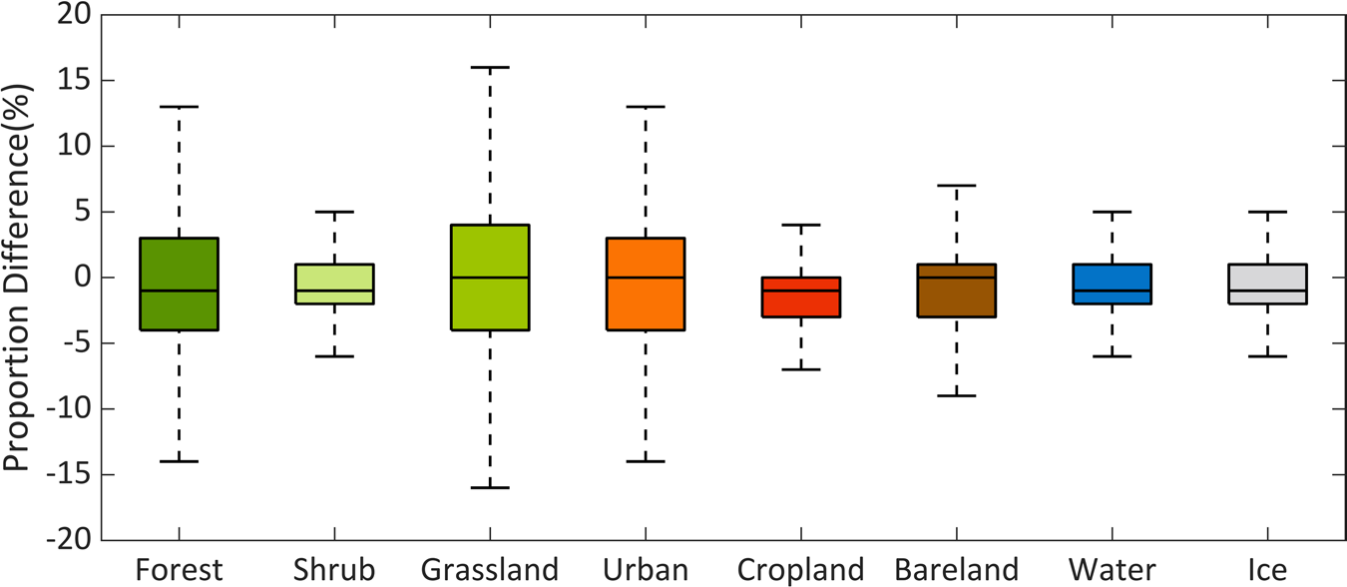
The difference in the land amount fractions of different LULC types within each 10-km x 10-km gird, which is calculated as our simulations minus the base map. For each boxplot, the boxes represent the interquartile ranges of the 25^th^ (Q_25_) and 75^th^ (Q_75_) percentiles, black line in each box represents the median value, and whiskers represent the values of Q_25_-1.5*(Q_75_-Q_25_) and Q_75_ + 1.5*(Q_75_-Q_25_). The color of each box corresponds to different LULC types.

We also compared the LULC spatial distributions of the actual LULC map retrieved by remote sensing data (CCI-LC, used as the base map), our downscaled data and LUH2 data in 2010 (Figure S3). It is worth noting that LUH2 data used a different historical data source^55^. Overall, they show similar spatial patterns with minor differences in most regions. The Pearson’s correlation coefficients between our data and base map are 0.98, 0.99, 0.95, 0.96, and 0.90 for Cropland, Forest, Bareland, Grassland and Urban. The differences between LUH2 and base map are mainly distributed in the eastern and south eastern China, where the base map shows a lower proportion of Bareland and Grassland, but a higher proportion of Urban. We further compared the difference of the land amount proportion of each LULC type within a 10-km x 10-km gird between our simulations and base map (Figure S4), which shows that the overall spatial pattern of our simulated LULC is consistent with the base map. Some differences between them are mainly distributed in the northwest of China, where our simulation overestimates the area of Bareland and underestimates the areas of Grassland and Cropland. This may be because from 2000 to 2010, some areas of Bareland were converted into Grassland and Cropland in the northwest China. However, our downscaling strategy assumes the Bareland can only be infringed by urban. Thus, the conversion from Bareland to other LULC types is omitted, which can lead to the overestimation of the areas of Bareland and underestimation of the area of Grassland and Cropland in this region. The fraction differences of all the eight LULC types within each 10-km × 10-km gird range from −15% to 15% and most of them are smaller than ± 5% (Fig. 2). All these results demonstrate that the downscaling process can accurately simulate the LULC spatial distributions.

### Spatio-temporal consistency with LUH2

We first analyzed the temporal change of the eight LULC types’ land amount in our dataset from 2010 to 2100 (Figure S5). Notably, Ice and Water are assumed to be intact in our LULC projections, and thus they will keep unchanged over time. Forest will increase in SSP1-, SSP2-, SSP4-based scenarios, but whether Forest increases or decreases in SSP3- and SSP5-based scenarios depends on specific RCP settings. Shrub shows an increasing trend in all the SSP scenarios. Grassland shows a decreasing trend in most of the SSP2-, SSP3-, SSP4-, and SSP5-based scenarios, a slight increase in the baseline scenarios of SSP4 and SSP5, and trivial changes in SSP1-based scenarios. Crop has a decreasing trend in all SSP1-, SSP4- and SSP5-based scenarios, but some SSP2- and SSP3-based scenarios show an increasing trend. In terms of RCP, more Croplands are required in RCP1.9, RCP2.6, and RCP3.4 than RCP4.5, RCP6, and baseline scenarios. Forest, Shrub, and Grassland will increase faster in RCP1.9, RCP2.6, and RCP3.4 than RCP4.5, RCP6, and baseline scenarios.

**Fig. 3:**
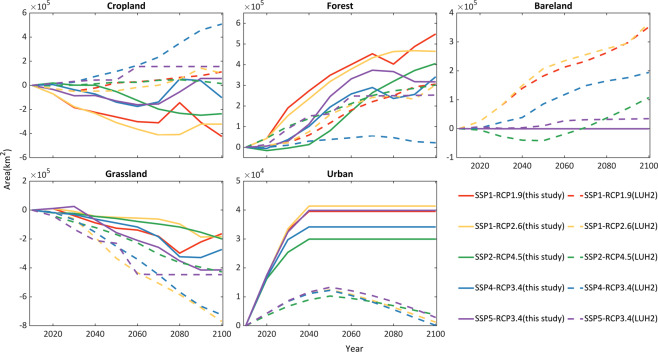
Comparison of the temporal trends of land areas for different LULC types between our dataset and LUH2. Here, land areas are represented by the change relative to the land area in 2010.

Then, we compared the LULC amounts of five overlapped scenarios between our dataset and LUH2 across the five LULC types. Figure 3 shows that the temporal trend of Forest is similar to LUH2 before 2060 and lightly differs from LUH2 after 2060, while the temporal trend of Grassland in our dataset is generally consistent with LUH2. Cropland in our dataset will decrease in all five scenarios, and presents different trends with LUH2. However, the trend of Cropland is similar with the results reported in Chen *et.al.* (2020b). The Urban shows an increasing trend from 2010 to the mid-21st century, consistent with LUH2. But Urban will remain stable after reaching its maximum values, which is different from the decreasing trends in LUH2. Bareland will slightly decrease from 2010 to mid-21st century and remain unchanged later, which is also different from LUH2. The difference in Urban and Bareland is mainly because these two types are assumed to be intact in GCAM, and we used an urban land dataset under SSPs to calibrate the future LULC change. This urban land dataset assumes that the urban will not convert to other LULC types in the city shrinking stage, which causes that the area of urban will keep unchanged after reaching the peak. Besides, we assume the Bareland can be infringed by urban in the expanding stage, leading to the slight decrease of Bareland from 2010 to the mid-21st century.

**Fig. 4:**
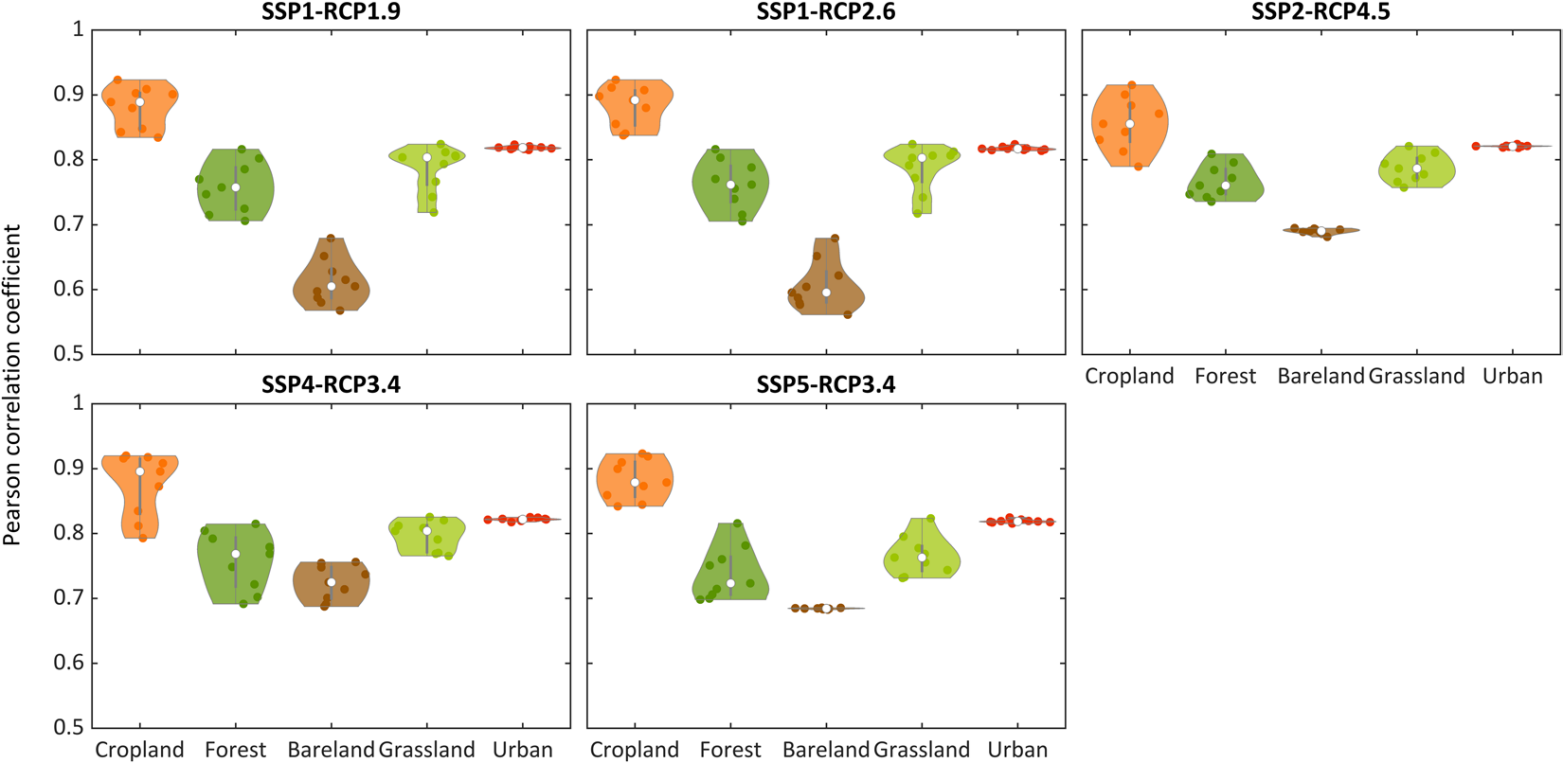
Comparison of the spatial consistencies between our downscaled gridded LULC dataset and LUH2 from 2010–2100. The points within each violin plot represent the calculated Person correlation coefficients between our data and LUH2 for all years.

We further compared the spatial consistencies of our downscaled gridded LULC dataset with LUH2 from 2010–2100 (Fig. 4). Overall, the two datasets show good consistencies across the five LULC types. The mean values of the Pearson’s correlation coefficients for five sceneries are 0.88 (Crop), 0.76 (Forest), 0.66 (Bareland), 0.78 (Grassland) and 0.82 (Urban) respectively. The relatively low consistency for Bareland may result from two reasons. First, the Bareland change in our dataset is caused by the urban encroachment since the demands generated by GCAM remain stable in the future, but LUH2 adopted different assumptions. Second, the base map we used had a distinct spatial pattern of Bareland compared to LUH2 in 2010 (see Figure S3). The Pearson’s correlation coefficients between our dataset and LUH2 range from 0.56 to 0.92 in SSP1-RCP1.9, 0.56 to 0.92 in SSP1-RCP2.6, 0.68 to 0.91 in SSP2-RCP3.4, 0.69 to 0.92 in SSP4-RCP3.4 and 0.68 to 0.92 in SSP5-RCP4.5. Among the five scenarios, our dataset has the highest Pearson’s correlation coefficients with LUH2 in SSP4-RCP3.4, which can be explained by that LUH2 used the same GCAM model with a different version (v4) for the LULC projection. These results demonstrate that our dataset has a good spatial consistency with LUH2 for different LULC types under different scenarios.

**Fig. 5:**
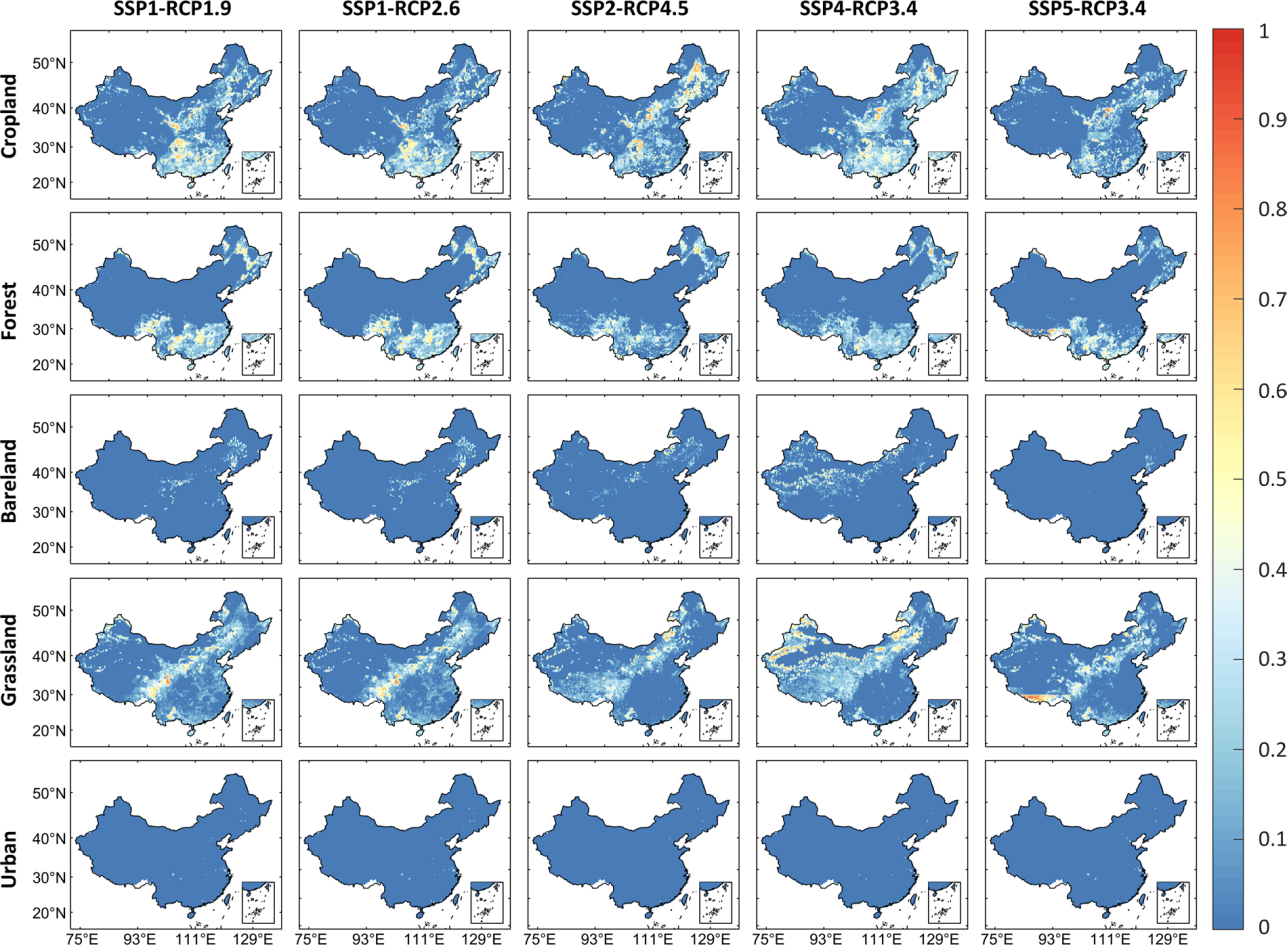
Comparison of the temporal consistencies (RMSD) of land area in five LULC types, calculated by our data minus LUH2. The blue and red colors indicate the low and high temporal differences between our data and LUH2, respectively.

We also compared the temporal consistencies (RMSD) of land area in five LULC types between our dataset and LUH2. As shown in Fig. 5, most of the temporal variation in our dataset is similar to LUH2, with only some minor differences. For all the five scenarios, the RMSD for Cropland ranges from 0.057 to 0.11; for Forest, it ranges from 0.052 to 0.073; for Bareland, it ranges from 0.001 to 0.026; for Grassland, it ranges from 0.062 to 0.11; and for Urban, it ranges from 0.0001 to 0.0006. Among all the LULC types, Urban shows the highest degree of similarity which may be because the urban dataset we used to calibrate the urban demand has a high consistency with LUH2^32^. But there are some differences in the temporal variation of Bareland and Grassland, and most of them occur in the northwest China, possibly because of the differences in the input data, the spatial downscaling strategy, and the new land cover classification scheme we used. The Bareland change is only caused by urban encroachment in our dataset, while this is not the case in LUH2. Some differences also occur in the southeast of China, which may result from the differences in type definition and classification standards between the base map and LUH2 (Figure S3). Overall our dataset shows high spatio-temporal consistency with LUH2. The discrepancy between our dataset and LUH2 can be caused by different IAM models, base map and LULC downscaling methods.

**Fig. 6:**
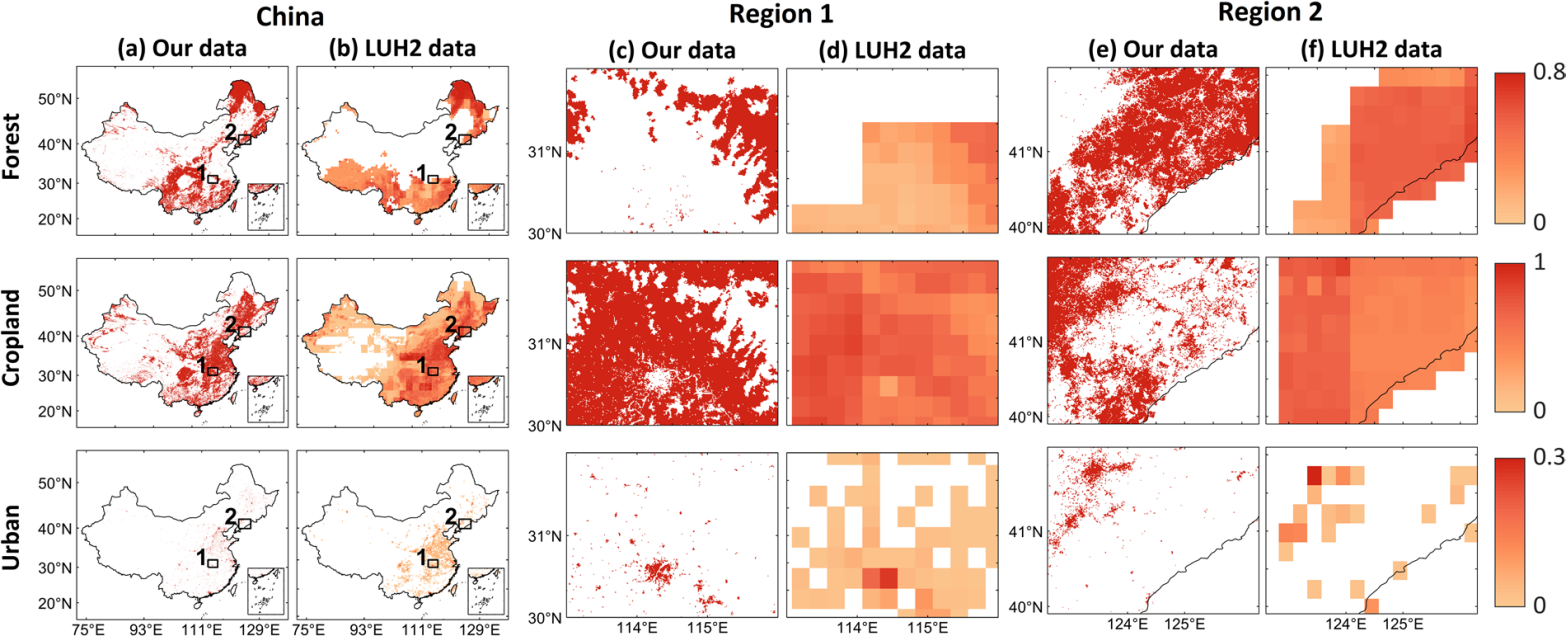
Comparison of the spatial details of Croplands, Forest and Urban between our dataset and LUH2 in SSP4-RCP3.4 in 2100 in China (**a**,**b**) and two typical regions: Region 1 (**c**,**d**) and Region 2 (**e**,**f**).

Besides, our dataset can reflect more spatial details because of its high spatial resolution (1-km) compared to LUH2 with coarse spatial resolution of 0.25°  (Fig. 6). This means our dataset can be more helpful to investigate the local impacts of LULC on ecosystem services and many other studies under different socioeconomic and emission conditions.

## Supplementary information

Supplementary material

## Data Availability

The GCAM v5.2 and FLUS models can be freely downloaded in https://github.com/JGCRI/gcam-core/releases and http://www.geosimulation.cn/FLUS.html, respectively.
