# Supplementary material for: 1 km land use/land cover change of China under comprehensive socioeconomic and climate scenarios for 2020–2100
Source: Sci Data. 2022 Mar 28;9:110. doi: 10.1038/s41597-022-01204-w (PMC8960815; doi:10.1038/s41597-022-01204-w)
Supplement: Supplementary file 1 — Supplementary material [file 41597_2022_1204_MOESM1_ESM.pdf]

Supplementary Information for

Gridded 1km Land Use Land Cover Change Projections of China Under Comprehensive SSP-RCP Scenarios

Meng Luo[1], Xia Li[1*], Guohua Hu[1*], Xiaojuan Liu[1], Hanyan Hou[1], Guangzhao Chen[2]

[1]Key Lab of Geographic Information Science (Ministry of Education), School of Geographic Sciences, East China Normal University, 500 Dongchuan Road, Shanghai 200241, China
[2] Institute of Future Cities,The Chinese University of Hong Kong, Shatin, NT, Hong Kong SAR.

Corresponding author: Xia Li (lixia@geo.ecnu.edu.cn), Guohua Hu (ghhu@geo.ecnu.edu.cn)

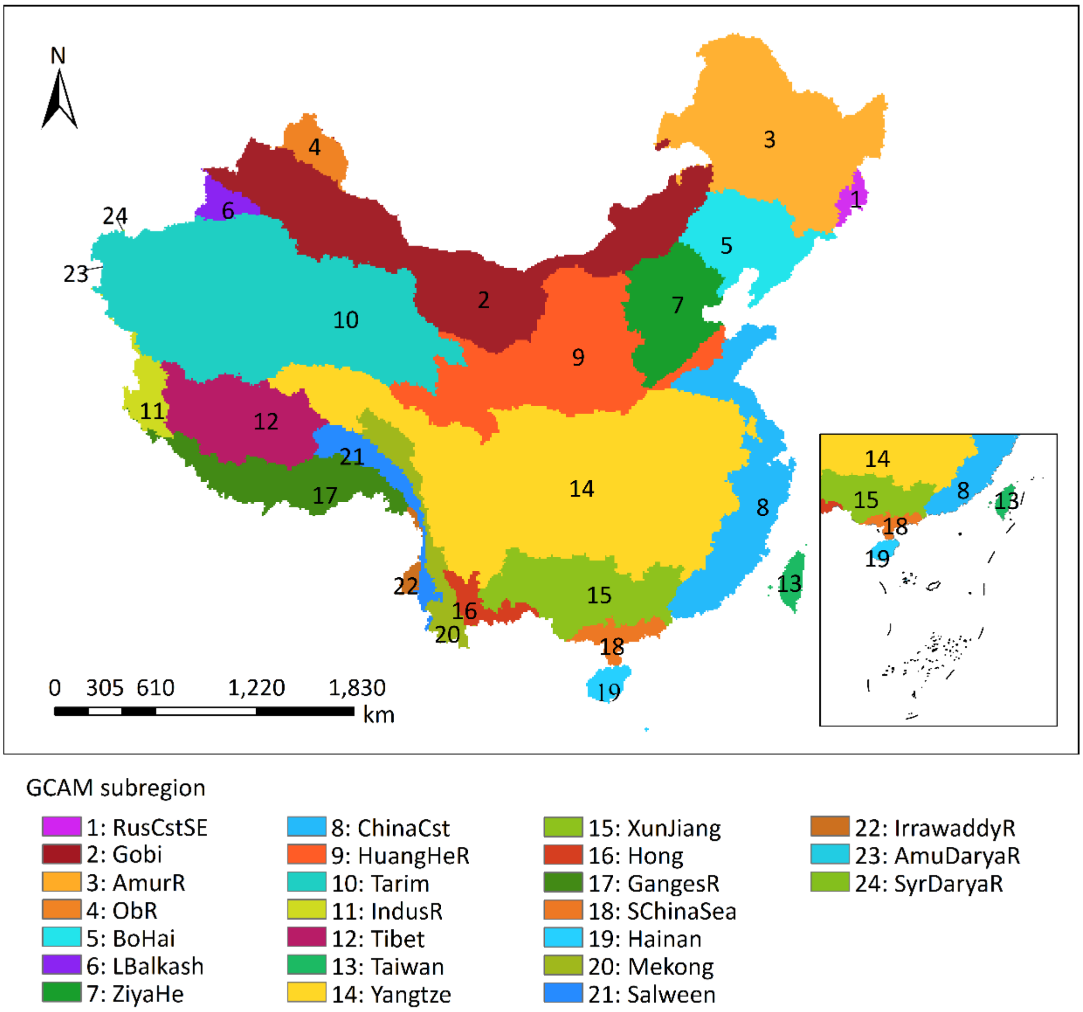

Figure S1. Spatial distribution of the 24 GCAM subregions in China.

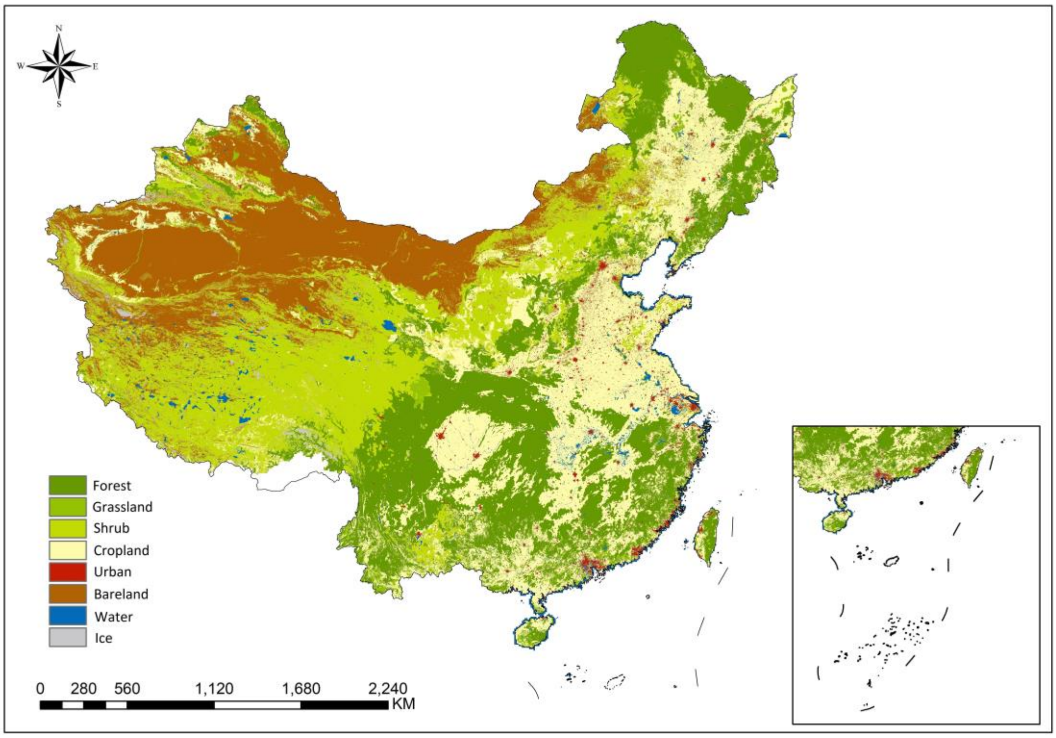

Figure S2. Spatial distribution of 1-km LULC types for 2100 under SSP1-RCP1.9 .

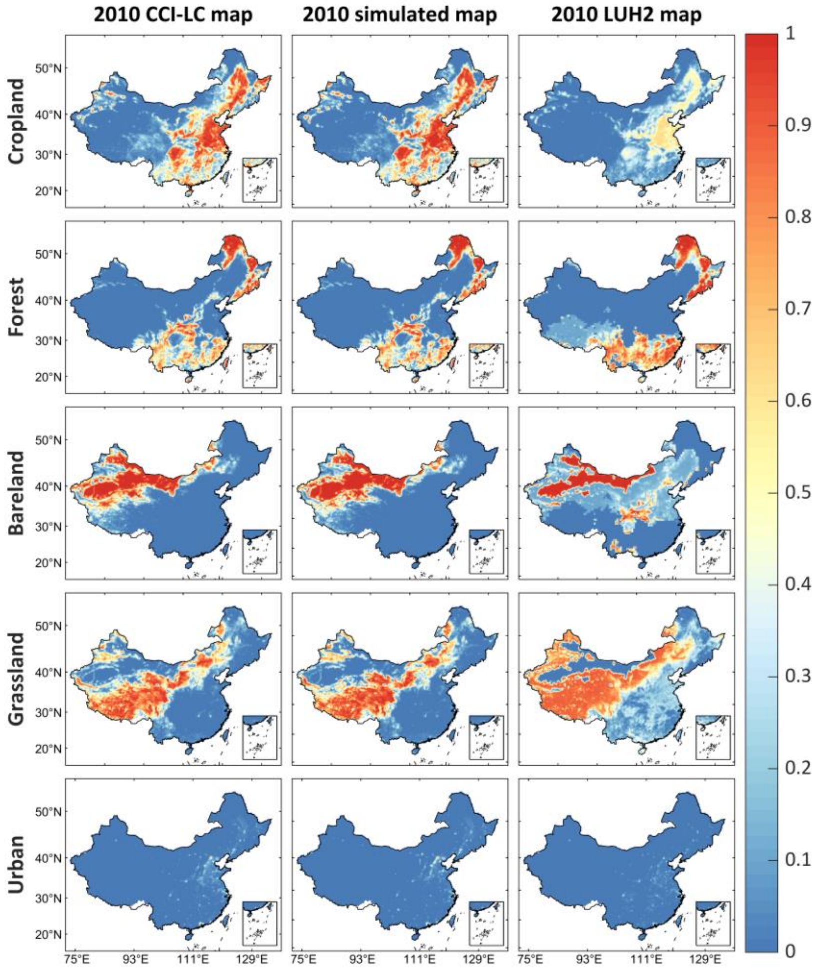

Figure S3. Comparison of the spatial distributions of LULC fractions among the actual LULC map retrieved by remote sensing data (CCI-LC, used as the base map), our downscaled data, and LUH2 data in 2010 within each 0.25˚×0.25˚ grid.

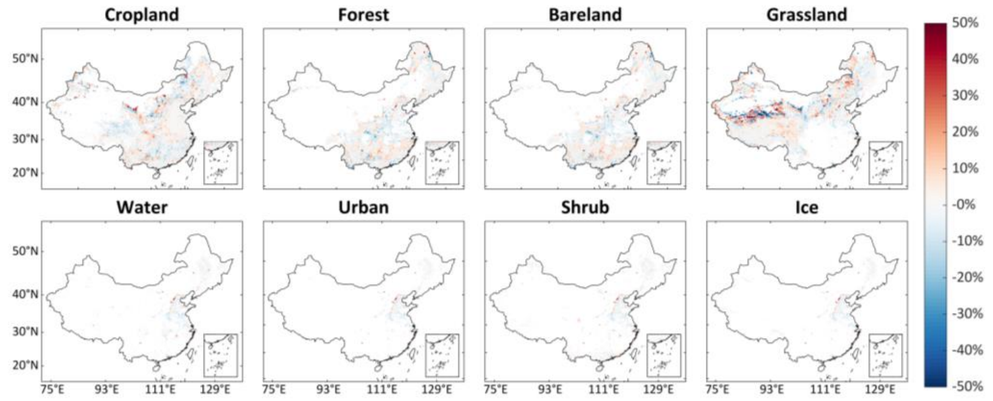

Figure S4. Spatial comparison of land demand fractions between our simulated LULC and base map within each 10-km x 10-km gird, calculated by simulated data minus base map in 2010. Red and blue colors represent overestimation and underestimation, respectively.

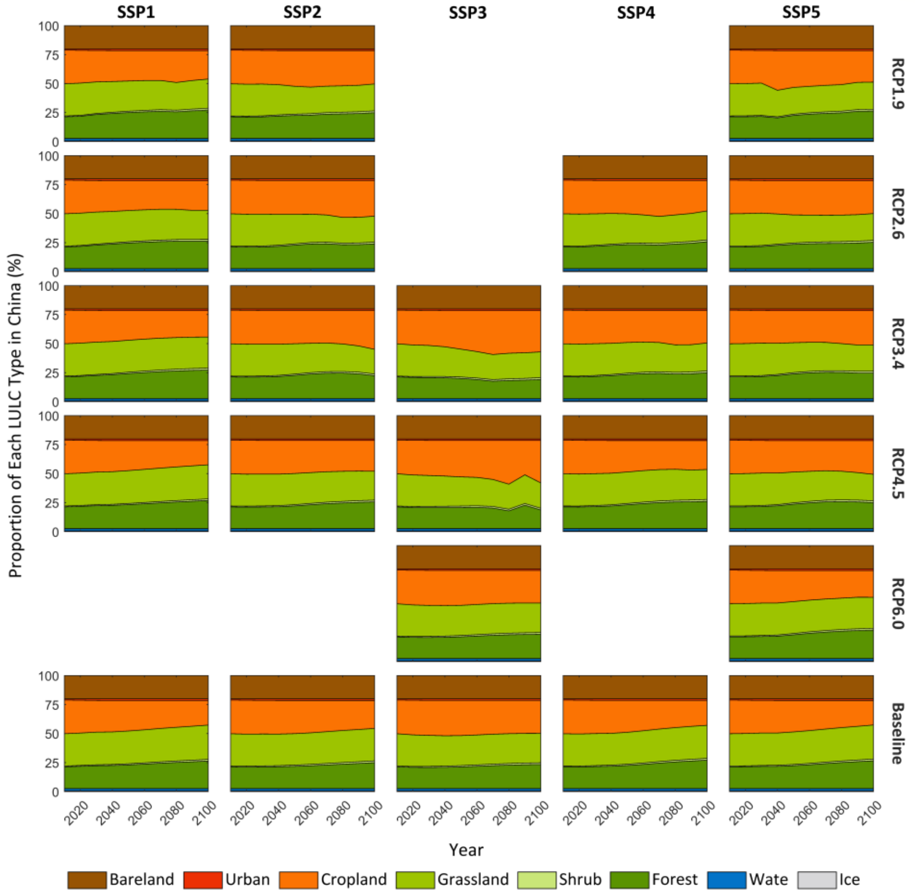

Figure S5. Temporal change of the land amount proportions of the eight LULC types in our data from 2010 to 2100.
